# TARGETING PARTICIPANTS AT HIGH RISK FOR ADVERSE HEALTH OUTCOMES IN THE R3 PROGRAM

**DOI:** 10.1093/geroni/igac059.822

**Published:** 2022-12-20

**Authors:** Marc Cohen, Edward Miller, Pamela Nadash

**Affiliations:** University of Massachusetts Boston, Boston, Massachusetts, United States; University of Massachusetts Boston, Boston, Massachusetts, United States; University of Massachusetts Boston, Boston, Massachusetts, United States

## Abstract

The R32 program seeks to ensure that individuals at high risk for adverse health outcomes are identified, engaged, and linked to needed services. The program pays particular attention to identifying individuals with risks related to mental health, memory, nutrition, food insecurity, and emergency department or inpatient hospitalizations. Key performance indicators were tracked, including the number and proportion of participants whose needs were addressed by R32. Results indicate that the program has succeeded in engaging the vast majority (>90%) of individuals with specific risk factors and connecting them with needed services. Viewed in the context of managed care plans, this level of performance is noteworthy and would earn the program a 5 Star rating – the highest rating available. Findings underscore the strong advantage offered by having a wellness nurse and wellness coordinator embedded on site in senior housing and using this platform to manage prevention and care services to residents.

